# Variation in perioperative practice in elective colorectal cancer surgery: opportunities for quality improvement

**DOI:** 10.1007/s12672-025-02254-3

**Published:** 2025-04-06

**Authors:** John C. Taylor, Hannah Rossington, Rina George, Sarah L. Alderson, Philip Quirke, Caroline Thomas, Simon Howell, the YCR BCIP Study Group

**Affiliations:** 1 Leeds Institute of Medical Research at St James’s, University of Leeds, Worsley Building, Leeds, LS2 9 NL UK; 2 Leeds Institute for Data Analytics, University of Leeds, Leeds, UK; 3 Doncaster and Bassetlaw Teaching Hospitals NHS Foundation Trust, Doncaster, UK; 4 Leeds Institute of Health Sciences, University of Leeds, Leeds, UK; 5 Leeds Teaching Hospitals NHS Trust, Leeds, UK

**Keywords:** Anaesthetics, Postoperative care, ERAS, Opioids, Preassessment, Multidisciplinary team

## Abstract

**Background:**

Understanding the variation in perioperative care across a population is fundamental to improving the management and outcomes of patients with colorectal cancer. Currently, there is limited individual patient level data available to assess this variation. Therefore, as part of an improvement programme, we conducted an audit to understand perioperative care.

**Methods:**

Audit items were developed to cover the pre, intra and postoperative phases of the colorectal cancer surgical pathway and collected for patients undergoing an elective procedure. The audit was conducted at 14 Hospital Trusts, participating in the Yorkshire Cancer Research Bowel Cancer Improvement Programme, located in the Yorkshire and Humber region, North of England.

**Results:**

Information on 216 patients were collected. Functional assessment by Cardiopulmonary Exercise Testing varied across the region (performed in 100% patients at three Trusts, but not at all in six Trusts, P < 0.001). The provision of postoperative high dependency and critical care also varied across the region (in seven Trusts ≥ 80% of patients went to a monitored bed or higher level of care; in three Trusts ≥ 90% of patients received ward care, P < 0.001). The median duration of preoperative starvation varied by Trust (2 to 13 h, P < 0.001). The intraoperative dose of opiate administered to patients varied significantly between Trusts (P < 0.001).

**Conclusions:**

There is wide variation in both the provision and practice of perioperative care across a large region in the North of England. The findings are informing a programme of improvement science-based work to improve the management and outcomes of patients with colorectal cancer.

**Supplementary Information:**

The online version contains supplementary material available at 10.1007/s12672-025-02254-3.

## Background

The objective of the Yorkshire Cancer Research funded Bowel Cancer Improvement Programme (YCR BCIP) is to improve survival from colorectal cancer across the Yorkshire and Humber in the north-east of England [1]. Outcomes from colorectal cancer in the region are comparable to those for the rest of the United Kingdom (UK) but not as good as elsewhere in Europe [2]. For example, 5-year net survival for colon cancer was lower in the UK (46%) than Denmark (64%), Norway (60%) and Ireland (51%) for the period 2010 to 2014 [3]. The region has a population of 5.7 million and 14 National Health Service (NHS) hospitals that deliver care for patients with colorectal cancer. The YCR BCIP program works to both support individual clinician practice and optimise local clinical systems.

Given that major surgical resection is the mainstay of treatment for patients with colorectal cancer, the perioperative care provided by hospitals may prove a key factor in patient outcomes. While a range of UK and international perioperative guideline exist, such as, those from the Centre for Perioperative Care (CPOC) [4], the Royal College of Anaesthetists (RCA) [5] and the Enhanced Recovery After Surgery (ERAS) Society [6], adherence to these within UK colorectal cancer services is not widely known. Adherence to perioperative protocols has been associated with improved postoperative outcomes following colorectal cancer surgery. Adherence to a standardised multimodal ERAS protocol significantly reduced postoperative 30-day morbidity, length of stay prolonging symptoms and some surgical complications [7]. A meta-analysis of randomised controlled studies in laparoscopic colorectal surgery found a significantly reduced postoperative length of hospital stay and complication rate with ERAS [8]. Additionally, non-adherence to ERAS following colon surgery was shown to be significantly associated with an increase in postoperative morbidity [9].

As in any healthcare system, the hospital Trusts and cancer multidisciplinary teams (MDTs) across Yorkshire vary in their size and the details of the patient pathway. The programme is, therefore, taking an approach based on improvement science through identifying local barriers and facilitators and supporting change through multilevel, multimodal and tailored implementation plans. A sprint audit provides rapidly available information designed to give a snapshot of services. The work described here was a sprint audit undertaken to understand perioperative care across the region, document variations in care and identify areas where practice can be harmonised or improved. Design of the audit was done through collaboration of surgical and anaesthetic colleagues to ensure the broad spectrum of key aspects in perioperative care was covered. Audit items covered the pre, intra and postoperative phases of the surgical pathway. We also collected routinely recorded data to understand the profile of the surgical population and the burden of adverse outcomes.

This work is intended to identify priorities for YCR BCIP and to provide a benchmark against which to evaluate changes in practice of perioperative care for patients with colorectal cancer. The audit was conducted during the COVID- 19 pandemic, but data were collected between waves of the pandemic and are likely to reflect usual practice.

## Methods

### Study population

The study population consisted of patients attending for surgery for colorectal cancer at any of the 14 Hospital Trusts within the YCR BCIP region (Yorkshire and the Humber). Only patients undergoing an elective procedure for colorectal cancer, with curative or palliative intent were included. Patients undergoing emergency surgery and those who had previously undergone an operation for colorectal cancer were excluded. The number of elective patients undergoing a major resection for a first diagnosed colorectal cancer per month were estimated using cancer registry and hospital admission data from the CORECT-R data repository [10]. Based on this, Trusts were grouped in small (< 8 cases per month), medium (8 to 12 cases per month) and large (> 12 cases per month) and set a target to recruit 10, 15 or 20 consecutive patients that matched the inclusion criteria respectively. This gave a total target of 205 cases.

### Data collection

Audit items to be included for data collection were finalised through a YCR BCIP working group consisting of anaesthetists and surgeons. Audit items covered the pre, intra and postoperative phases of the surgical pathway. To be included, an item had to reflect a key aspect of perioperative care, be amenable to change, and ideally be the subject of national or international recommendations to guide best practice [4–6]. A pilot audit using an early draft of the data collection tool was conducted in three hospitals. The tool was then refined to avoid ambiguity and guidance was written to support the data collection process. The final 44 item data collection form was developed to facilitate substantial data retrieval while taking into consideration the clinical time pressures of the teams involved. The audit tool was distributed to the colorectal cancer MDT in each hospital and colleagues were identified to support the collection of anaesthetic and surgical data. Data were collected via paper form (Supplementary Methods) between May 2021 and February 2022 and the 44 non-identifiable items were submitted to a central online collection tool omitting any patient identifiers (onlinesurverys.ac.uk).

### Variables studied

The full collection tool and audit response options can be found in the Supplementary Information. Variables describing patient characteristics included age at operation, body mass index (BMI), American Society of Anesthesiologists Physical Status Classification (ASA Grade), co-existing cardiac disease, respiratory disease and renal impairment. Frailty was assessed using the Clinical Frailty Scale [11]. The presence of the following potentially modifiable risk factors was recorded: alcohol consumption, current smoking, diet, anaemia and low levels of activity.

Preoperative variables included type of preassessment, use of Cardiopulmonary Exercise Testing (CPEX/CPET) functional assessment, preoperative starvation time and type of bowel prep given prior to surgery. The risk of surgery was assessed by the anaesthetist and surgeon, and on the basis of the functional assessment. High-risk was defined as predicted hospital mortality ≥ 5% following the assessment.

Intraoperative variables included whether management was done by a consultant anaesthetist with a regular practice in colorectal cancer or not, duration of surgery, whether an unplanned transfusion took place, management with goal directed fluid therapy, and use of beat-to-beat blood pressure monitoring, cardiac output or stroke volume variability monitoring, siting of a central venous catheter and general anaesthetic, regional/neuraxial anaesthesia and system analgesia. Use of an opioid sparing anaesthetic was defined as the use of nonopioid adjuvant medication and regional anaesthesia, including peripheral and neuraxial nerve blocks as a perioperative strategy to decease opioid use [12]. The type and dose of opiates given during surgery was recorded and converted to morphine equivalents for analysis [13].

Postoperative variables included use of multimodal postoperative analgesia, if the patient was seen by a member of the acute pain team and if an ERAS nurse was involvement in postoperative care/present at postoperative destination. Markers of postoperative outcomes included total length of stay in hospital, whether there was a surgical site infection, acute kidney injury (classified using the 2012 KDIGO criteria [14], postoperative complications (classified using the Clavien-Dindo scale [15]), an unplanned return to theatre and discharge destination.

### Statistics

We mainly used descriptive analyses using proportions, medians and interquartile range (IQR) to assess the variation between Trusts for each item collected. Differences in patient characteristics and differences in practice between Trusts were tested using Fisher’s exact test or using a non-parametric test on the equality of medians. A non-parametric test on the equality of median was used to test association between morphine equivalent dose and opiate sparing technique. Missing or unknown categories were excluded from the statistical tests. A *p*-value of < 0.05 was considered statistically significant. All analyses were performed using Stata (16.1, StataCorp LLC, College Station, TX).

## Results

### Patient characteristics

All 14 Trusts (4 small, 7 medium and 3 large) invited to participate submitted cases to the study. The total number of cases submitted was 216. Between Trusts, there was no significant difference in age (median 70.0 years, IQR = 11.0, P = 0.206), BMI (median 27.7 kg m^−2^, IQR = 7.7 P = 0.720), ASA grade (P = 0.311) and planned surgery (P = 0.264). Only 4% of patients were reported to be at least mildly frail (CFS ≥ 5) (Table 1, Supplementary Figure S1). The most frequently reported potentially modifiable risk factor was anaemia (21% of patients) and there was evidence of a significant difference in the number of modifiable risk factors between Trusts (P = 0.003).

**Table 1:** Patient characteristics of the study population

Characteristic	Category	N	% or IQR	Trust difference p-value^a^
Total cases		216		
Age	Median (IQR)	70.0	63.0–74.0	0.206
BMI	Median (IQR)	27.7	24.3–32.0	0.720
ASA Grade	1 or 2	136	63.0	0.311
	3 or 4	77	35.6	
	Unknown	3	1.4	
Frailty (CFS)	Very Fit to Managing Well	166	76.9	0.037
	Vulnerable	15	6.9	
	Mildly frail to Severely Frail	9	4.2	
	Unknown	26	12.0	
Comorbidity	Present	131	60.7	< 0.001
	None	83	38.4	
	Unknown	2	0.9	
Modifiable Risk Factors	None	76	35.2	< 0.001
	1	61	28.2	
	≥ 2	77	35.7	
	Unknown	2	0.9	

^a^ P-values from Fisher’s exact test or non-parametric test on the equality of medians

### Preoperative Assessment

**Fig. 1:**
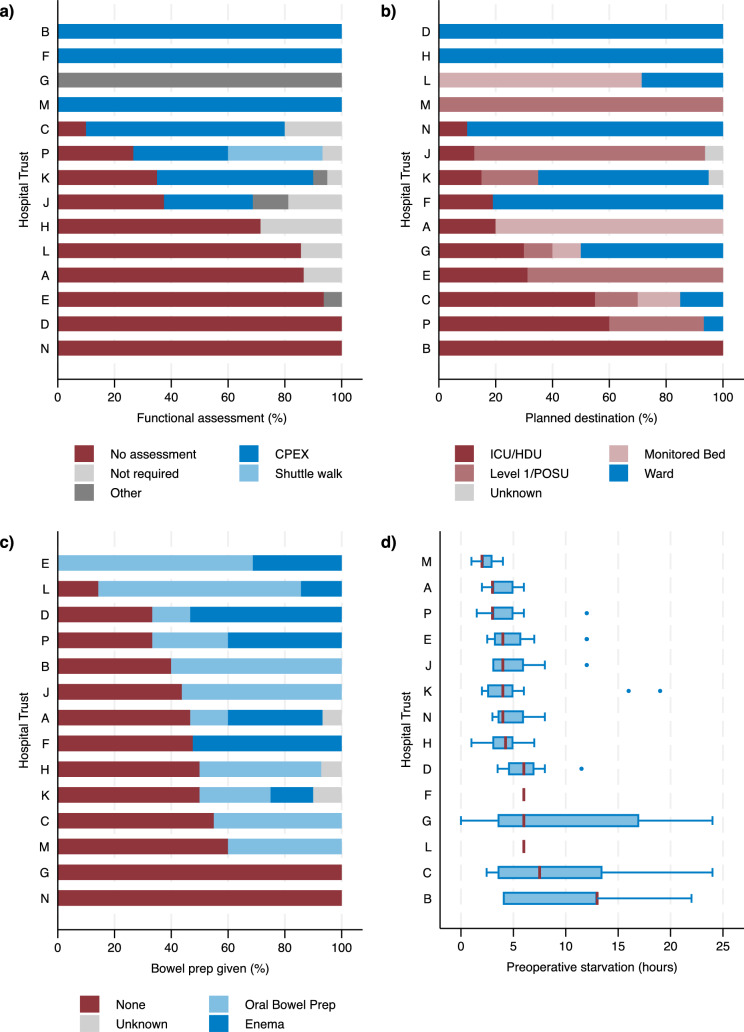
Bar charts (% of patients) and Tukey boxplots showing variation by Hospital Trust (A-P) in selected preoperative items for **a** type of functional assessment (CPEX = cardiopulmonary exercise Testing), **b** planned level of postoperative destination (ICU = intensive care unit, HDU = high dependency unit, POSU = postoperative surgical unit), c) type of bowel preparation given and d) length of preoperative starvation

A functional assessment was performed on 102 (47%) patients and this significantly varied across Trusts (P < 0.001, Table 2). CPEX testing was performed in 100% patients at three Trusts but not at all in 6 of the Trusts (Fig. 1). Although 21 patients were aged 80 years or older, no assessments by elderly care were performed (Supplementary Table S1).

**Table 2:** Results for selected pathway items

Item	Category	N	% or IQR	Trust difference p-value^a^
*Preoperative pathway*				
Functional assessment	Assessment performed	102	47.2	< 0.001
	No assessment	85	39.4	
	Not required or unknown	29	13.4	
Bowel prep given	Yes	112	51.8	< 0.001
	No	100	46.3	
	Unknown	4	1.9	
Planned destination	ICU, HDU or Level 1/POSU	108	50.0	< 0.001
	Monitored bed or Ward	106	49.1	
	Unknown	2	0.9	
Preop starvation (hours)	Median (IQR)	4.5	3.0–6.0	< 0.001
*Intraoperative pathway*				
Duration of surgery (hours)	Median (IQR)	3.5	2.8–4.8	< 0.001
Intraoperative Management	Colorectal anaesthetist	126	58.3	< 0.001
	Other	87	40.3	
	Unknown	3	1.4	
General anaesthetic	Volatile	131	60.7	< 0.001
	TIVA	83	38.4	
	Unknown	2	0.9	
Equivalent IV morphine dose (mg)	Median (IQR)	10.0	0.0–14.0	< 0.001
*Postoperative pathway*				
Length of stay (days)	Median (IQR)	7.0	4.0–10.0	0.023
Postoperative complication	Yes	98	45.4	0.021
	No	116	53.7	
	Unknown	2	0.9	
Acute pain team	Yes	90	41.7	< 0.001
	No	123	56.9	
	Unknown	3	1.4	
ERAS nurse at destination	No	143	66.2	< 0.001
	Yes	62	28.7	
	Unknown	11	5.1	

^a^ P-values from Fisher’s exact test or non-parametric test on the equality of medians

One hundred (46%) of patients had no preoperative bowel preparation and this varied significantly across Trusts (P < 0.001, Table 2), 33% underwent oral bowel prep and 19% received an enema (Fig. 1, Supplementary Table S1).

The planned destination after surgery varied significantly by Trust, with some exclusively planning to use HDU and others exclusively a ward (P < 0.001, Table 2, Fig. 1). The median duration of preoperative starvation varied significantly by Trust from 2 to 13 h (P < 0.001, Table 2, Fig. 1). Overall, 52 (8%) patients were assessed as being high risk status in either the surgeon or anaesthetic assessment.

### Intraoperative items

**Fig. 2:**
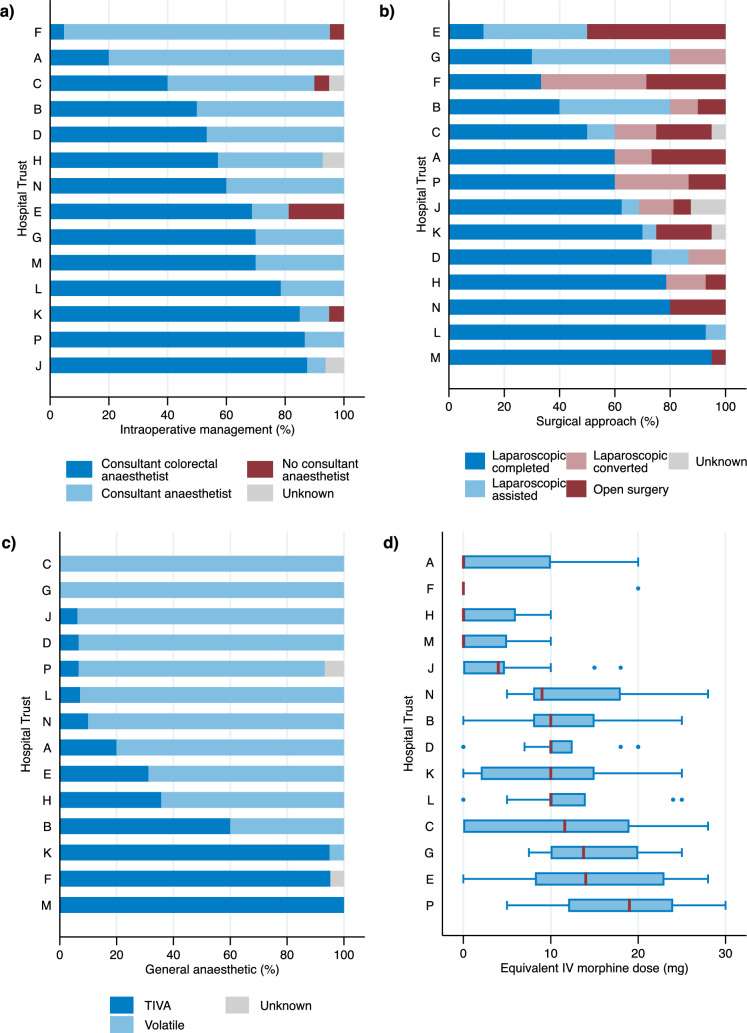
Bar charts (% of patients) and Tukey boxplots showing variation by Hospital Trust (A-P) in selected intraoperative items for **a** intraoperative management, **b** surgical approach, **c** general anaesthetic given (TIVA = total intravenous anaesthesia), and **d** equivalent intravenous (IV) dose given

Management by a colorectal anaesthetist was done in 126 (58%) of patients overall and varied significantly by Trust from 5 to 88% (P < 0.001, Table 2, Fig. 2).

Volatile general anaesthetics were used in the 131 (61%) of patients compared to TIVA in 83 (38%), and significantly varied by Trust (P < 0.001, Table 2, Fig. 2). Spinal anaesthesia was used in 140 (65%) of all patients overall, and in 100% of patients at two Trusts. Epidurals (15%), TAP blocks (14%) and rectus sheath blocks (11%) were used more sparingly but were used at least 50% of the time in some Trusts (Supplementary Table S2).

An opiate sparing anaesthetic technique was said to be used in three quarters of patients (Supplementary Table S2). However, there was no difference in the morphine equivalent dose between the patients recorded as having an opiate sparing technique used (median = 10.0, IQR = 0–13.2) or not used (median, 10.0, IQR = 4.0–15.0), p = 0.408. The total intraoperative dose of opiate, in morphine equivalents, varied significantly between Trusts (P < 0.001, Table 2, Fig. 2).

Overall, 184 (85%) patients were given paracetamol in the intraoperative period, and it was used at least 50% of the time in all Trusts (Supplementary Table 2, Supplementary Fig. 2). The use of other non-opiate analgesics was more variable. Magnesium and ketamine were each used in approximately a quarter of patients, clonidine and intravenous lidocaine were used in less than 10% of patients.

### Postoperative items

**Fig. 3:**
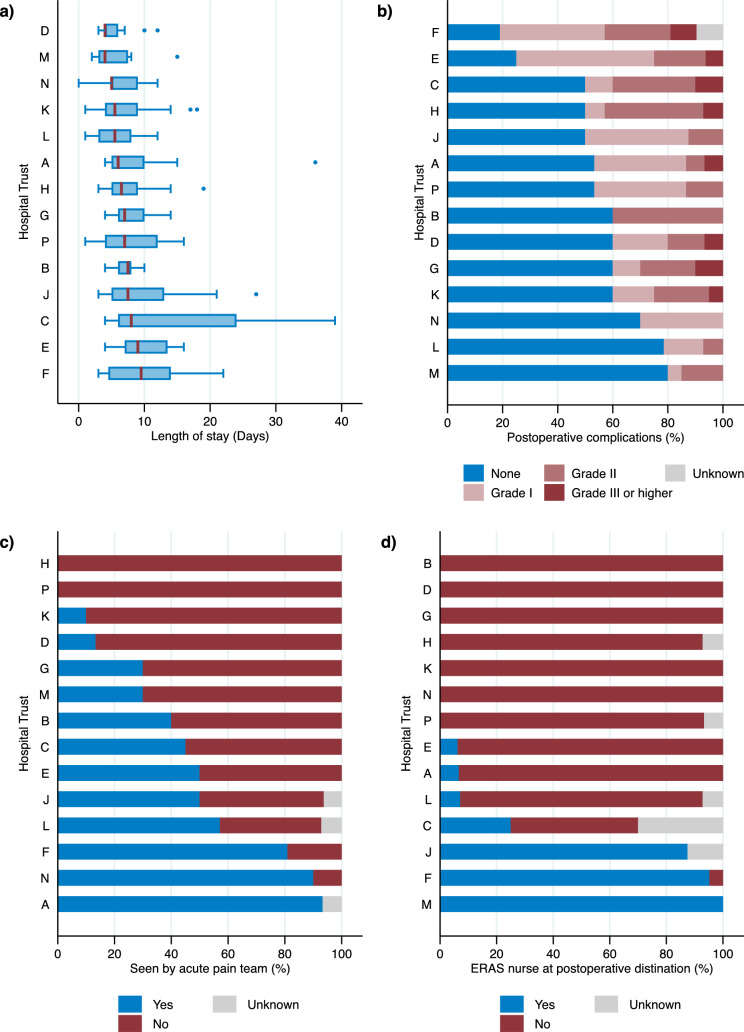
Bar charts (% of patients) and Tukey boxplots showing variation by Hospital Trust (A-P) in postoperative items for **a** length of patient stay after surgery, **b** postoperative complications, **c** if the patient was seen by an acute pain team, and d) if an enhanced recovery after surgery (ERAS) nurse was present at the postoperative destination

The median length of stay ranged from 4 to 10 days across Trusts (P = 0.023, Table 2, Fig. 3). A total of 98 (45%) patients experienced a postoperative complication of Grade I or higher (Table 2, Fig. 3), however only 6 (3%) patients required a return to theatre (Supplementary Figure S3.).

No patients at two of the Trusts were seen by an acute pain team, but at least 80% of patients were seen by a team in three of the Trusts (P < 0.001, Supplementary Table S3 and Figure S3). An ERAS nurse was not in place at the postoperative destination for all patients in 7 of the Trusts but was in place for most patients at 3 of the Trusts (P < 0.001, Table 2, Fig. 3).

## Discussion

This study demonstrated wide variation in both the provision and practice of perioperative care across a large region in the North of England with a population of over 5.5 million people. The work was conducted in the context of a large programme of research aimed at improving the care and outcomes from colorectal cancer patients across the Yorkshire region. With this in view, the results were used to identify opportunities to improve perioperative care and surgical outcomes. This discussion focuses on those areas with the most immediate potential for change that will improve both the patient experience and outcomes from colorectal cancer. These include provision of functional testing, provision of high dependency and critical care, a reduction in preoperative starvation times, adherence to bowel preparation guidelines and opioid prescribing practice.

The provision of functional assessment to identify and optimise treatment of higher risk patients was variable across the region. Reduced functional capacity is associated with an increased risk of perioperative complications [16]. The UK CPOC guidelines recommend that all patients being considered for surgery should be screened for reduced functional capacity and those with reduced capacity on screening should undergo objective assessment with cardiopulmonary exercise testing or a similar test [17]. The provision of preoperative CPEX and similar testing was highly variable. Three hospitals undertook CPEX testing on all patients, six offered objective functional testing to very few or none of the patients in the current survey. Formal testing of all patients may not be necessary, but our data suggest that there is under provision or underuse of preoperative functional capacity assessment in region.

Current guidelines recommend that all patients being considered for surgery should undergo an individualised risk assessment. Those with a greater than 1% risk of postoperative mortality should be considered for enhanced postoperative care and those with a greater than 5% risk should be considered for postoperative critical care admission [4]. The provision of postoperative high dependency and critical care was highly variable. In seven hospitals 80% or more of patients went to a monitored bed or high level of care. In three hospitals 90% or more of patients received ward care. At a system and hospital level, systematic provision of higher levels of postoperative care (e.g. high dependency) for high-risk patients is needed. The provision of critical and high-acuity care beds in the UK is significantly less than many other developed countries. For example, in 2018 it was reported that the estimated per capita high-acuity bed capacities per 100,000 population were 1.2, 3.8, and 6.4 in the UK, Australia, and NZ, respectively [18]. It is now recognised that older patients being considered for surgery, and particularly those that are frail, benefit from specialist multidisciplinary care [19]. However, our data indicate that there is very limited specific provision for the assessment and management of the older patient being considered for colorectal surgery. Our group has demonstrated that fewer elderly patients receive surgery for colorectal cancer than that in Denmark [20]. The reasons for this are the subject of further ongoing work. As the population ages this issue will become steadily more pressing.

The evidence of both discomfort and harm from long-periods of deprivation of food and fluids before surgery is well documented. Initiatives reduce preoperative starvation have had limited success. This is significant part due to the constraints of ensuring the best use of operating theatres in an environment where resources are constrained, and the order of the operating list may have to be changed at short notice [21]. Anecdotally, in the current study patients who were planned to have the most major operations were more likely to undergo prolonged starvation because the start of surgery was deferred until an intensive care or high-dependency bed was confirmed to be available. The challenge of prolonged preoperative starvation and our changing understanding of the risks of aspiration of gastric contents has led to the development of “Sip til send” policies for patients undergoing fractured hip surgery [22]. This approach, whereby the patient can take measured amounts of water by mouth until the time they go to the operating theatre, is gradually being expanded to other types of surgery. It has the potential to significantly reduce starvation times for colorectal surgery patients across Yorkshire.

There was slight variability in preoperative bowel preparation protocols. The Association of Coloproctology issued guidance in 2017 stating that mechanical bowel preparation is not routinely recommended for colonic resection [23]. It may, however, be considered in restorative rectal cancer surgery. In our cohort, one Trust stood out as giving all resectional patients mechanical bowel preparation. This may be considered out of line with national practice. In the majority, the use of mechanical bowel preparation was reserved for rectal cancer surgery. Most administered a pre-operative phosphate enema for left sided resections, however, one Trust was noted to not give any pre-operative bowel preparation to their patients.

On the one hand opioids are invaluable both for providing strong analgesia as part of balanced anaesthesia in many settings and for postoperative pain relief; on the other the adverse effects of opioids (e.g. nausea, vomiting, sedation, ileus) may significantly slow recovery. Should opioids need to be continued into the postoperative period and beyond to discharge they can be associated with significant patient harm (dependency and addiction) and increased mortality, especially if patient becomes established on ongoing treatment with a modified release opioid [24, 25]. The ERAS guidelines advocate the use of multimodal opioid sparing analgesia, combining the use of several non-opioid analgesic treatments to reduce both the intraoperative and postoperative use of opioids [6]. Whilst an opioid sparing anaesthetic technique was said to be have been used in 75% of patients in this study, in practice the dose of opioids administered was variable. The widespread use of paracetamol and spinal analgesia was encouraging but the use of other opiate sparing drugs was limited. The use of a formal protocol for postoperative analgesia and access to an acute pain team in the postoperative period was variable and likely increased the probability of patients being discharged home with a new prescription for opioid drugs.

A limitation of this study is the relatively small sample size from each Hospital Trust. A larger sample size would have allowed significance testing between process and outcomes. However, the primary aim of the study was to assess variation across hospitals and the target recruitment numbers were based on a pragmatic decision covering hospital size and the capacity to collect data while performing usual clinical duties.

This study demonstrates several areas where there is potential for quality improvement initiatives to improve patient experience, outcomes, and survival from colorectal cancer within Yorkshire and, by extrapolation, across the UK. The YCR BCIP programme aims to support and bring about changes to practice at the individual patient and practitioner level. Examples of these include a local expert group on opioid prescribing has been established and is developing a toolkit of quality indicators to underpin change in opioid prescribing practice. A similar approach is likely to be applicable to reducing preoperative starvation and the rational use of mechanical bowel preparation. The provision of targeted multidisciplinary care for older surgical patients would likely improve outcomes in the region. Within YCR BCIP work is in progress to examine the impact of frailty on the progress of patients down the treatment pathway from diagnosis to both medical and surgical treatment. In parallel to this, is work to improve processes within individual Trusts, to improve screening for frailty early in the patient pathway.

In summary, this study of the perioperative care of patients with colorectal cancer in Yorkshire and the Humber has yielded valuable information about the provision of surgical care at both the system and the individual patient level applicable within the region and likely beyond. The findings are informing a programme of improvement science-based work to improve the management and outcomes of patients with colorectal cancer.

## Supplementary Information

Additional file 1. Supplementary Tables S1-S3, Supplementary Figure S1-S3.

Additional file 2. Supplementary Methods.

## Data Availability

The data supporting the results of the sprint audit are available from the corresponding author upon reasonable request. The cancer registry data used in the sample size calculations of this study are available through CORECT‐R and NHS Digital subject to relevant approvals, they are not available directly from the authors.

## References

[1] Taylor J, Wright P, Rossington H, Mara J, Glover A, West N, Morris E, Quirke P. group YBs: Regional multidisciplinary team intervention programme to improve colorectal cancer outcomes: study protocol for the Yorkshire Cancer Research Bowel Cancer Improvement Programme (YCR BCIP). BMJ Open. 2019;9(11): e030618. 31772088 10.1136/bmjopen-2019-030618 PMC6886907

[2] Allemani C, Matsuda T, Di Carlo V, Harewood R, Matz M, Niksic M, Bonaventure A, Valkov M, Johnson CJ, Esteve J, et al. Global surveillance of trends in cancer survival 2000–14 (CONCORD-3): analysis of individual records for 37 513 025 patients diagnosed with one of 18 cancers from 322 population-based registries in 71 countries. Lancet. 2018;391(10125):1023–75. 29395269 10.1016/S0140-6736(17)33326-3 PMC5879496

[3] Arnold M, Rutherford MJ, Bardot A, Ferlay J, Andersson TM, Myklebust TA, Tervonen H, Thursfield V, Ransom D, Shack L, et al. Progress in cancer survival, mortality, and incidence in seven high-income countries 1995–2014 (ICBP SURVMARK-2): a population-based study. Lancet Oncol. 2019;20(11):1493–505. 31521509 10.1016/S1470-2045(19)30456-5 PMC6838671

[4] Centre for Perioperative Care (CPOC). Preoperative assessment and optimisation for adult surgery. 2021 [https://www.cpoc.org.uk/guidelines-resources/guidelines]

[5] Royal College of Anaesthetists. Guidelines for the Provision of Anaesthesia Services. 2022 [https://rcoa.ac.uk/safety-standards-quality/guidance-resources/guidelines-provision-anaesthetic-services]

[6] Gustafsson UO, Scott MJ, Hubner M, Nygren J, Demartines N, Francis N, Rockall TA, Young-Fadok TM, Hill AG, Soop M, et al. Guidelines for Perioperative Care in Elective Colorectal Surgery: Enhanced Recovery After Surgery (ERAS((R))) Society Recommendations: 2018. World J Surg. 2019;43(3):659–95. 30426190 10.1007/s00268-018-4844-y

[7] Gustafsson UO, Hausel J, Thorell A, Ljungqvist O, Soop M, Nygren J. Enhanced Recovery After Surgery Study G: Adherence to the enhanced recovery after surgery protocol and outcomes after colorectal cancer surgery. Arch Surg. 2011;146(5):571–7. 21242424 10.1001/archsurg.2010.309

[8] Ni X, Jia D, Chen Y, Wang L, Suo J. Is the Enhanced Recovery After Surgery (ERAS) Program Effective and Safe in Laparoscopic Colorectal Cancer Surgery? A Meta-Analysis of Randomized Controlled Trials. J Gastrointest Surg. 2019;23(7):1502–12. 30859422 10.1007/s11605-019-04170-8

[9] Ceresoli M, Pedrazzani C, Pellegrino L, Ficari F, Braga M. Perioperative Italian Society study g: Early non compliance to enhanced recovery pathway might be an alert for underlying complications following colon surgery. Eur J Surg Oncol. 2024;50(5): 106650. 35817632 10.1016/j.ejso.2022.06.033

[10] Downing A, Hall P, Birch R, Lemmon E, Affleck P, Rossington H, Boldison E, Ewart P, Morris EJA. Data Resource Profile: The COloRECTal cancer data repository (CORECT-R). Int J Epidemiol. 2021;50(5):1418–1418k. 34255059 10.1093/ije/dyab122 PMC8580263

[11] Rockwood K, Song X, MacKnight C, Bergman H, Hogan DB, McDowell I, Mitnitski A. A global clinical measure of fitness and frailty in elderly people. CMAJ. 2005;173(5):489–95. 16129869 10.1503/cmaj.050051 PMC1188185

[12] Kumar K, Kirksey MA, Duong S, Wu CL. A review of opioid-sparing modalities in perioperative pain management: methods to decrease opioid use postoperatively. Anesth Analg. 2017;125(5):1749–60. 29049119 10.1213/ANE.0000000000002497

[13] Arnold R, Weissman DE. Calculating opioid dose conversions #36. J Palliat Med. 2003;6(4):619–20. 14516504 10.1089/109662103768253731

[14] Goren O, Matot I: Perioperative acute kidney injury. *Br J Anaesth* 2015, 115 Suppl 2:ii3–14. 10.1093/bja/aev380 26658199

[15] Dindo D, Demartines N, Clavien PA. Classification of surgical complications: a new proposal with evaluation in a cohort of 6336 patients and results of a survey. Ann Surg. 2004;240(2):205–13. 15273542 10.1097/01.sla.0000133083.54934.ae PMC1360123

[16] Moran J, Wilson F, Guinan E, McCormick P, Hussey J, Moriarty J. Role of cardiopulmonary exercise testing as a risk-assessment method in patients undergoing intra-abdominal surgery: a systematic review. Br J Anaesth. 2016;116(2):177–91. 26787788 10.1093/bja/aev454

[17] Preoperative Assessment and Optimisation for Adult Surgery including consideration of COVID-19 and its implications, Centre for Perioperative Care [https://www.cpoc.org.uk/preoperative-assessment-and-optimisation-adult-surgery]

[18] Wong DJN, Popham S, Wilson AM, Barneto LM, Lindsay HA, Farmer L, Saunders D, Wallace S, Campbell D, Myles PS, et al. Postoperative critical care and high-acuity care provision in the United Kingdom, Australia, and New Zealand. Br J Anaesth. 2019;122(4):460–9. 30857602 10.1016/j.bja.2018.12.026 PMC6435907

[19] Partridge JS, Harari D, Martin FC, Peacock JL, Bell R, Mohammed A, Dhesi JK. Randomized clinical trial of comprehensive geriatric assessment and optimization in vascular surgery. Br J Surg. 2017;104(6):679–87. 28198997 10.1002/bjs.10459

[20] Taylor JC, Iversen LH, Burke D, Finan PJ, Howell S, Pedersen L, Iles MM, Morris EJA, Quirke P, Group YBS. Influence of age on surgical treatment and postoperative outcomes of patients with colorectal cancer in Denmark and Yorkshire. England Colorectal Dis. 2021;23:3152–61. 10.1111/codi.15910 34523211

[21] Hewson DW, Moppett I: Preoperative fasting and prevention of pulmonary aspiration in adults: research feast, quality improvement famine. *Br J Anaesth* 2020. 10.1016/j.bja.2019.12.018 31980163

[22] Scottish Hip Fracture Audit Steering Group. Developing ‘SipTilSend’ policies for hip fracture surgery. 2022 [https://www.shfa.scot.nhs.uk/Resources/index.html]

[23] Moran B, Cunningham C, Singh T, Sagar P, Bradbury J, Geh I, Karandikar S. Association of Coloproctology of Great Britain & Ireland (ACPGBI): Guidelines for the Management of Cancer of the Colon, Rectum and Anus (2017) - Surgical Management. Colorectal Dis. 2017;19(Suppl 1):18–36. 28632309 10.1111/codi.13704

[24] Levy N, Quinlan J, El-Boghdadly K, Fawcett WJ, Agarwal V, Bastable RB, Cox FJ, de Boer HD, Dowdy SC, Hattingh K, et al. An international multidisciplinary consensus statement on the prevention of opioid-related harm in adult surgical patients. Anaesthesia. 2021;76(4):520–36. 33027841 10.1111/anae.15262

[25] Quinlan J, Levy N, Lobo DN, Macintyre PE. No place for routine use of modified-release opioids in postoperative pain management. Br J Anaesth. 2022;129(3):290–3. 35843745 10.1016/j.bja.2022.06.013

