# Supplementary material for: Variation in perioperative practice in elective colorectal cancer surgery: opportunities for quality improvement
Source: Discov Oncol. 2025 Apr 6;16:473. doi: 10.1007/s12672-025-02254-3 (PMC11972997; doi:10.1007/s12672-025-02254-3)
Supplement: Supplementary file 1 — Additional file 1. Supplementary Tables S1-S3, Supplementary Figure S1-S3. [file 12672_2025_2254_MOESM1_ESM.docx]

**Supplementary Material – Supplementary Tables and Figures**

**Supplementary Table S1.** Full results from the preoperative items

| **Preoperative Item** |  | **N** | **%** | **Trust median range (%)** |
| --- | --- | --- | --- | --- |
| Preassessment* | Preop visit (nurse/AHP) | 158 | 73.2 | 15.0 – 100 |
|  | Clinic visit (anaesthetist) | 77 | 35.7 | 0 – 100 |
|  | Telephone assessment (nurse/AHP) | 48 | 22.2 | 0 – 90.0 |
|  | Assessment by elderly care | 0 | 0 | 0 – 0 |
|  | Notes review only | 2 | 0.9 | 0 – 13.3 |
| Functional assessment | No assessment | 94 | 43.5 | 0 – 100 |
|  | CPEX | 86 | 39.8 | 0 – 100 |
|  | Shuttle walk | 5 | 2.3 | 0 – 33.3 |
|  | Other | 14 | 6.5 | 0 – 100 |
|  | Not required | 17 | 7.9 | 0 – 28.6 |
| High risk (functional) | No | 124 | 57.4 | 0 – 100 |
|  | Yes | 23 | 10.7 | 0 – 45.0 |
|  | Not documented | 69 | 31.9 | 0 – 100 |
| High risk (anaesthetic) | No | 139 | 64.4 | 15.0 – 100 |
|  | Yes | 34 | 15.8 | 0 – 50.0 |
|  | Not documented | 43 | 19.9 | 0 – 80.0 |
| High risk (surgeon) | No | 122 | 56.5 | 0 – 100 |
|  | Yes | 18 | 8.3 | 0 – 31.3 |
|  | Not documented | 76 | 35.2 | 0 – 100 |
| Bowel prep given | None | 100 | 46.3 | 0 – 100 |
|  | Oral | 72 | 33.3 | 0 – 71.4 |
|  | Enema | 40 | 18.5 | 0 – 53.3 |
|  | Unknown | 4 | 1.9 | 0 – 10.0 |
| Planned destination | ICU/HDU | 51 | 23.6 | 0 – 100 |
|  | Level 1/POSU | 57 | 26.4 | 0 – 100 |
|  | Monitored bed | 26 | 12.0 | 0 – 80.0 |
|  | Ward | 80 | 37.0 | 0 – 100 |
|  | Unknown | 2 | 0.9 | 0 – 6.3 |
| Preop starvation (hours) | Median (IQR) | 4.5 | 3.0 – 6.0 | 2.0 – 13.0 |

* Not mutually exclusive

**Supplementary Table S2.** Full results from the intraoperative items

| **Intraoperative Item** |  | **N** | **%** | **Trust median range (%)** |
| --- | --- | --- | --- | --- |
| Surgical approach | Laparoscopic completed | 130 | 60.2 | 12.5 – 95.0 |
|  | Laparoscopic assisted | 22 | 10.2 | 0 – 50.0 |
|  | Laparoscopic converted | 26 | 12.0 | 0 – 38.1 |
|  | Open | 34 | 15.7 | 0 – 50.0 |
|  | Unknown | 4 | 1.9 | 0 – 12.5 |
| Duration of surgery (hours) | Median (IQR) | 3.5 | 2.8 – 4.8 | 2.3 – 5.0 |
| Intraoperative Management | Consultant colorectal anaesthetist | 126 | 58.3 | 4.8 – 87.5 |
|  | Consultant anaesthetist | 81 | 37.5 | 6.3 – 90.5 |
|  | No consultant anaesthetist | 6 | 2.8 | 0 – 18.8 |
|  | Unknown | 3 | 1.4 | 0 – 7.1 |
| Cardiovascular management | Beat to beat blood pressure monitoring | 138 | 63.9 | 0 – 100 |
|  | Cardiac output or stroke volume monitoring | 94 | 43.5 | 0 – 73.3 |
|  | Managed by goal directed fluid therapy | 95 | 44.0 | 0 – 100 |
|  | Central venous catheter sited | 14 | 6.5 | 0 – 23.8 |
|  | Unplanned blood transfusion | 9 | 4.2 | 0 – 33.3 |
| General anaesthetic | Volatile | 131 | 60.7 | 0 – 100 |
|  | TIVA | 83 | 38.4 | 0 – 100 |
|  | Unknown | 2 | 0.9 | 0 – 6.7 |
| Regional Anaesthesia* | Spinal | 140 | 64.8 | 0 – 100 |
|  | Epidural | 33 | 15.3 | 0 – 57.1 |
|  | TAP | 31 | 14.4 | 0 – 55.0 |
|  | Rectus sheath | 23 | 10.7 | 0 – 50.0 |
|  | Other regional | 7 | 3.2 | 0 – 12.5 |
|  | Other | 22 | 10.2 | 0 – 80.0 |
| Systemic analgesia* | Paracetamol | 184 | 85.2 | 50.0 – 100 |
|  | Magnesium | 61 | 28.2 | 0 – 100 |
|  | Ketamine | 52 | 24.1 | 0 – 100 |
|  | NSAIDs | 42 | 19.4 | 0 – 50.0 |
|  | Lidocaine | 19 | 8.8 | 0 – 60.0 |
|  | Clonidine | 15 | 6.9 | 0 – 35.0 |
|  | Other | 18 | 8.3 | 0 – 70.0 |
| Opioid sparing technique | Use reported | 158 | 73.2 | 0 – 100 |
| Opioids used*† | IV Fentanyl | 85 | 39.4 | 0 – 100 |
|  | IV Morphine | 58 | 26.9 | 0 – 100 |
|  | IV Remifentanil | 49 | 22.7 | 0 – 100 |
|  | IV Oxycodone | 27 | 12.5 | 0 – 43.8 |
|  | Intrathecal Diamorphine | 109 | 50.5 | 0 – 100 |
|  | Intrathecal preservative-free Morphine | 29 | 13.4 | 0 – 93.3 |
| Equivalent IV morphine dose (mg) | Median (IQR) | 10.0 | 0.0 – 14.0 | 0 – 19.0 |

* Not mutually exclusive; † Those used in >10% patients

**Supplementary Table S3.** Full results from the postoperative items

| **Postoperative Item** |  | **N** | **%** | **Trust median range (%)** |
| --- | --- | --- | --- | --- |
| Immediate destination | ICU/HDU | 65 | 30.1 | 0 – 100 |
|  | Level 1/POSU | 53 | 24.5 | 0 – 95.0 |
|  | Monitored bed | 21 | 9.7 | 0 – 80.0 |
|  | Ward | 76 | 35.2 | 0 – 100 |
|  | Unknown | 1 | 0.5 | 0 – 10.0 |
| Length of stay | Median (IQR) | 7.0 | 4.0 – 10.0 | 4.0 – 10.0 |
| Seen by acute pain team on day 1 | No | 123 | 56.9 | 0 – 100 |
|  | Yes | 90 | 41.7 | 0 – 93.3 |
|  | Unknown | 3 | 1.4 | 0 – 7.1 |
| Surgical site infection | No | 191 | 88.4 | 66.7 – 100 |
|  | Yes | 22 | 10.2 | 0 – 33.3 |
|  | Unknown | 3 | 1.4 | 0 – 7.1 |
| Acute kidney injury | No | 201 | 93.1 | 76.2 – 100 |
|  | Yes | 12 | 5.6 | 0 – 23.8 |
|  | Unknown | 3 | 1.4 | 0 – 6.3 |
| Postoperative complication (CD) | None | 116 | 53.7 | 19.1 – 80.0 |
|  | Grade I | 48 | 22.2 | 0 – 50.0 |
|  | Grade II | 40 | 18.5 | 0 – 40.0 |
|  | Grade III – Grade V | 10 | 4.6 | 0 – 10.0 |
|  | Unknown | 2 | 0.9 | 0 – 9.5 |
| Unplanned return to theatre | No | 205 | 94.9 | 87.5 – 100 |
|  | Yes | 6 | 2.8 | 0 – 9.5 |
|  | Unknown | 5 | 2.3 | 0 – 18.8 |
| Mortality at point of discharge | Alive | 213 | 98.6 | 87.5 – 100 |
|  | Died | 1 | 0.5 | 0 – 4.8 |
|  | Unknown | 2 | 0.9 | 0 – 12.5 |
| Started therapeutic antibiotics | No | 160 | 74.1 | 37.5 – 100 |
|  | Yes | 53 | 24.5 | 0 – 56.3 |
|  | Unknown | 3 | 1.4 | 0 – 6.3 |
| Discharge destination | Usual residence and level of care | 208 | 96.3 | 90.5 – 100 |
|  | Increased level of care/support | 6 | 2.8 | 0 – 20.0 |
|  | Unknown | 2 | 0.9 | 0 – 6.7 |
| ERAS nurse involved in care | No | 31 | 14.4 | 0 – 73.3 |
|  | Yes | 71 | 32.9 | 0 – 100 |
|  | N/A No ERAS nurse | 105 | 48.6 | 0 – 100 |
|  | Unknown | 9 | 4.2 | 0 – 25.0 |
| ERA nurse at destination | No | 143 | 66.2 | 0 – 100 |
|  | Yes | 62 | 28.7 | 0 – 100 |
|  | Unknown | 11 | 5.1 | 0 – 30.0 |

**Supplementary Figure S1.** Bar charts (% of patients) and Tukey boxplots showing variation by Hospital Trust (A-P) in selected patient characteristics for a) age in years, b) body mass index, c) ASA grade and d) Clinical Frailty Scale.

**Supplementary Figure S2.** Bar charts (% of patients) showing variation by Hospital Trust (A-P) in use of non-opiate analgesics

**Supplementary Figure S3.** Bar charts (% of patients) showing variation by Hospital Trust (A-P) in selected postoperative items
