# Supplementary material for: Variation in perioperative practice in elective colorectal cancer surgery: opportunities for quality improvement
Source: Discov Oncol. 2025 Apr 6;16:473. doi: 10.1007/s12672-025-02254-3 (PMC11972997; doi:10.1007/s12672-025-02254-3)
Supplement: Supplementary file 2 — Additional file 2. Supplementary Methods. [file 12672_2025_2254_MOESM2_ESM.pdf]

Supplementary Material – Supplementary Methods

Below are copies of the collection form (pages 1-2) and field information guides (pages 3-8) which were provided to the collection teams. Collection was done by paper form and uploaded to a central online database.

YCRBCIP – Anaesthetics and Surgery Sprint Audit 2021

**A. Patient Name**	Click or tap here to enter text.		
**B. Patient NHS Number**	Click or tap here to enter text.		
**1. Local Hospital Audit Ref Number**			
**2. Age**	Click or tap here to enter text.		
**3. BMI**	Click or tap here to enter text.		
**4. Planned Operation**	Choose an item.		
	If other please specify:		
**5. Is the operation planned as open or laparoscopic?**	Open ☐ Laparoscopic ☐		
**6. Type of Pre-Assessment** (select all options that apply)	Pre-Op Visit (face to face nurse or AHP) ☐<br>Clinic Visit (face to face anaesthetist) ☐<br>Telephone Assessment (nurse or AHP) ☐<br>Assessment by Elderly Care(face to face or telephone) ☐<br>Notes review only – no additional assessment ☐		
**7. Meet Criteria for Functional Assessment**<br>Choose an item.	**8. Functional Assessment Performed**<br>Choose an item.	**9. ASA**<br>Choose an item.	
**10. Frailty** (using clinical frailty scale)	Choose an item.		
**11. High Risk (Functional Assessment)**<br>Choose an item.	**12. High Risk (anaesthetics assessment)**<br>Choose an item.	**13. High Risk (surgeon assessment)**<br>Choose an item.	
**14. Modifiable Risk factors identified** (tick all that apply)	Alcohol ☐ Smoking☐ Anaemia☐ Diet☐<br>Respiratory ☐ Cardiac☐ Diabetes☐ Activity☐ Renal☐<br>Unknown☐<br>No☐		
**15. Pre-Op Starvation Duration from last oral intake (food or fluid) in hours**	Click or tap here to enter text.		
**16. Bowel Prep Given**	Choose an item.		
**17. Actual operation:**	Choose an item.		
	If other please specify:		
**18. Was the actual operation laparoscopic or open?**	Laparoscopic completed ☐<br>Laparscopic converted to open ☐<br>Laparoscopic assisted ☐<br>Open ☐		
**19. Duration of surgery** (hours/ minutes – knife to skin to completion)	Click or tap here to enter text.		
**20. Unplanned intraoperative transfusion**<br>Choose an item.	**21. Intraoperative management by consultant anaesthetist**<br>Choose an item.	**22. Managed by goal directed fluid therapy**<br>Choose an item.	**23. Opioid sparing technique**<br>Choose an item.
**24. Regional or neuraxial anaesthesia**	Spinal ☐ Epidural ☐ TAP☐<br>Rectus Sheath ☐ Other Regional ☐<br>Specify Other: Click or tap here to enter text.		

YCRBCIP Data Collection Form for Perioperative Sprint Audit 2021 v1.1 05 May 2021

<table>
<tr><td>25. Systemic analgesia</td><td colspan="8">IV Lidocaine ☐ IV Clonidine ☐ IV Ketamine ☐
IV Magnesium ☐ Paracetamol ☐ NSAIDS ☐
Gabapentin ☐ Other ☐</td></tr>
<tr><td rowspan="2">26. Intra-Operative Opioid Use</td><td>IV Fentanyl
µg</td><td>IV Morphine
mg</td><td>Intrathecal Fentanyl
µg</td><td>Intrathecal Diamorphine
µg</td><td>Intrathecal Preservative-Free Morphine
µg</td><td>Epidural Morphine
mg</td><td>Epidural Fentanyl
µg</td><td>Other (Specify drug and dose)</td></tr>
<tr><td></td><td></td><td></td><td></td><td></td><td></td><td></td><td></td></tr>
<tr><td>27. Type of General Anaesthetic</td><td colspan="8">Choose an item.</td></tr>
<tr><td>28. Beat to Beat Blood Pressure Monitoring
Choose an item.</td><td colspan="4">29. Cardiac output or stroke volume variability monitoring
Choose an item.</td><td colspan="4">30. Central Venous Catheter Sited
Choose an item.</td></tr>
<tr><td>31. Immediate Postop destination Planned (pre-op)</td><td colspan="8">Choose an item.</td></tr>
<tr><td>32. Immediate Postop destination Actual</td><td colspan="8">Choose an item.</td></tr>
<tr><td>33. If available, was protocolised postop multimodal analgesia prescribed?</td><td colspan="8">Choose an item.</td></tr>
<tr><td>34. Was the patient seen by the acute pain team on day one post-operative?</td><td colspan="8">Choose an item.</td></tr>
<tr><td>35. Was ERAS Nurse involved in post-operative care?</td><td colspan="8">Choose an item.</td></tr>
<tr><td>36. Length of Stay (days)</td><td colspan="8">Click or tap here to enter text.</td></tr>
<tr><td>37. Surgical Site Infection</td><td colspan="8">Choose an item.</td></tr>
<tr><td>38. AKI</td><td colspan="8">Choose an item.</td></tr>
<tr><td>39. Post-Operative Complications (Clavien-Dindo)</td><td colspan="8">Choose an item.</td></tr>
<tr><td>40. Unplanned Return to theatre with this admission
Choose an item.</td><td colspan="3">41. Mortality at point of discharge
Choose an item.</td><td colspan="2">42. ERAS nurse present at post-operative destination
Choose an item.</td><td colspan="3">43. Started post-operative therapeutic antibiotic
Choose an item.</td></tr>
<tr><td>44. Discharge Destination from Hospital</td><td colspan="8">Choose an item.</td></tr>
</table>

Field Number	Field Name	Definition	Requirements for data field completion	Suggested person collecting/ providing this data item
A.	Patient Name	Patient's Name.	Alphanumeric field.<br>For local use only. Not to transferred to University of Leeds	Either
B.	Patient NHS Number	Patient's NHS Number	Numeric Field.<br>For local use only. Not to transferred to University of Leeds	Either
1.	Local Hospital Audit Reference Number	This should be a unique number assigned by the Hospital. If there are queries with any data submitted this will be the number quoted by the University of Leeds. Hospitals must keep a local record of the audit numbers assigned to each patient if not kept on the data collection form.<br>It must not be the patient's NHS number or hospital number.	Alphanumeric field.	Either
2.	Age	Age of patient on date of surgery	Numeric field.	Anaesthetist
3.	BMI	The BMI of patient as assessed prior to surgery	Numeric field.	Anaesthetist

Field Number	Field Name	Definition	Requirements for data field completion	Suggested person collecting/ providing this data item
4.	Planned operation	Operation Type planned prior to surgery.	Select one option only<br>Panproctocolectomy<br>proctocolectomy and ileo-anal pouch<br>total colectomy and ileorectal anastomosis<br>total colectomy unspecified<br>extended right hemicolectomy<br>right hemicolectomy<br>transverse colectomy<br>left hemicolectomy<br>sigmoid colectomy<br>abdomino-perineal resection of rectum<br>anterior resection with anastomosis<br>Hartmann's procedure<br>colostomy only<br>ileostomy only<br>TEMS<br>TART<br>Endoscopic excision of malignant polyp<br>Colonic stent<br>Pelvic exenteration<br>Other (please specify)	Surgeon
5.	Is the operation planned as open or laparoscopic?	Confirm if the approach to the operation is planned as an open or laparoscopic approach.	Select one option only<br>Open<br>Laparoscopic	Surgeon

Field Number	Field Name	Definition	Requirements for data field completion	Suggested person collecting/ providing this data item
6.	Type of Pre-Assessment	Type of Pre-assessment completed.	Select all options that apply<br>Pre-Op Visit (face to face nurse or AHP)<br>Clinic Visit (face to face anaesthetist)<br>Telephone Assessment (nurse or AHP)<br>Assessment by Elderly Care(face to face or telephone)<br>Notes review only – no additional assessment	Anaesthetist
7.	Meet Criteria for Functional Assessment	Does the patient meet the criteria for functional assessment as specified by the audit?<br>For the purposes of this audit, the suggested criteria for functional assessment are:<br>• if the patient is aged >55 years, OR is <55 years with one of the following comorbidities:<br>• Ischaemic heart disease<br>• Heart failure<br>• Valvular heart disease<br>• Diabetes<br>• COPD/Asthma<br>• Chronic kidney disease<br>• Chronic liver disease	Select one option only.<br>Yes<br>No	Anaesthetist
8.	Functional Assessment Performed	The type of functional assessment has been performed	Select one option only.<br>Not Required<br>No<br>Shuttle Walk<br>CPEX<br>Other	Anaesthetist
9.	ASA	ASA Grade of patient assessed prior to surgery	Select one option only.<br>1/ 2/ 3/ 4	Anaesthetist

Field Number	Field Name	Definition	Requirements for data field completion	Suggested person collecting/ providing this data item
10	Frailty (using clinical frailty scale)	Frailty assessed in accordance with the enclosed clinical frailty scale (see page 13 for scale).	Select one option only.<br>Very Fit<br>Well<br>Managing Well<br>Vulnerable<br>Mildly Frail<br>Moderately Frail<br>Severely Frail<br>Very Severely Frail<br>Terminally Ill	Anaesthetist
11.	High Risk (Functional Assessment)	Has the patient been assessed as high risk (predicted hospital mortality ≥5%) following functional assessment?	Select one option only.<br>Yes<br>No<br>Not documented	Anaesthetist
12.	High Risk (Anaesthetics Assessment)	Has the patient been assessed as high risk (predicted hospital mortality ≥5%) following anaesthetist's assessment?	Select one option only.<br>Yes<br>No<br>Not documented	Anaesthetist
13.	High Risk (Surgeon Assessment)	Has the patient been assessed as high risk (predicted hospital mortality ≥5%) following surgeon's assessment?	Select one option only.<br>Yes<br>No<br>Not documented	Surgeon

Field Number	Field Name	Definition	Requirements for data field completion	Suggested person collecting/ providing this data item
14.	Modifiable Risk Factors identified	Have any modifiable risk factors been identified?<br>Please note this field refers to risk factors that have been identified only. It is not necessary for any actions to have taken place to modify the risk factor.	Select all that apply<br>Alcohol<br>Smoking<br>Anaemia<br>Diet<br>Respiratory<br>Cardiac<br>Diabetes<br>Activity<br>Renal<br>Unknown<br>None Identified	Anaesthetist
15.	Pre-Op Starvation Duration from last oral intake (food or fluid)	The total duration of pre-operative starvation from the patient's last oral intake whether this was food or fluid in hours. Note this includes carbohydrate drinks.	Numeric field in hours	Anaesthetist
16.	Bowel Prep Given	The type of bowel prep the patient has been given prior to surgery	Select one option only<br>None<br>Oral Bowel Prep<br>Enema	Surgeon

Field Number	Field Name	Definition	Requirements for data field completion	Suggested person collecting/ providing this data item
17.	Actual Operation	The actual operation completed.	Select one option from list<br>Same as planned<br>Panproctocolectomy<br>proctocolectomy and ileo-anal pouch<br>total colectomy and ileorectal anastomosis<br>total colectomy unspecified<br>extended right hemicolectomy<br>right hemicolectomy<br>transverse colectomy<br>left hemicolectomy<br>sigmoid colectomy<br>abdomino-perineal resection of rectum<br>anterior resection with anastomosis<br>Hartmann's procedure<br>colostomy only<br>ileostomy only<br>TEMS<br>TART<br>Endoscopic excision of malignant polyp<br>Colonic stent<br>Pelvic exenteration<br>Other (please specify)	Surgeon
18.	Was the actual operation laparoscopic or open?	State the actual approach to the operation that took place	Select one option<br>Laparoscopic completed<br>Laparscopic converted to open<br>Laparoscopic assisted<br>Open	Surgeon
19.	Duration of Surgery	Knife to skin to completion of surgery	Alphanumeric field<br>To be recorded in hours and minutes	Surgeon

Field Number	Field Name	Definition	Requirements for data field completion	Suggested person collecting/ providing this data item
20.	Unplanned intraoperative transfusion	Did an unplanned intraoperative transfusion take place?	Select one option<br>Yes<br>No	Anaesthetist
21.	Intraoperative management by consultant anaesthetist	Was the patient managed intraoperatively by a consultant anaesthetist with a regular practice in colorectal cancer?	Select one option<br>Yes – consultant anaesthetist<br>Yes – consultant anaesthetists with regular colorectal sessions<br>No	Anaesthetist
22.	Managed by goal directed fluid therapy	Has the patient been managed in accordance with goal directed therapy?	Select one option<br>Yes<br>No	Anaesthetist
23.	Opioid Sparing technique	Was an opioid sparing anaesthetic technique used?<br>Defined as use of nonopioid adjuvant medication and regional anesthesia, including peripheral and neuraxial nerve blocks as a perioperative strategy to decrease opioid use.<br>This is based on *Kumar, Kanupriya MD* et al. ‡ A Review of Opioid-Sparing Modalities in Perioperative Pain Management: Methods to Decrease Opioid Use Postoperatively, Anesthesia & Analgesia 2017; 125: 1749-1760*	Select one option<br>Yes<br>No	Anaesthetist
24.	Regional or neuraxial anaesthesia	The type (if any) of regional or neuraxial technique that was used during the operation	Select the option(s) that applies<br>Spinal<br>Epidural<br>TAP<br>Rectus Sheath<br>Other regional (please specify)<br>Specify Other (enter text)	Anaesthetist

Field Number	Field Name	Definition	Requirements for data field completion	Suggested person collecting/ providing this data item
25.	Systemic analgesia	The type of analgesia that was used during the operation	Select the option(s) that applies<br>IV Lidocaine<br>IV Clonidine<br>IV Ketamine<br>IV Magnesium<br>Paracetamol<br>NSAIDS<br>Gabapentin<br>Specify Other (enter text)	Anaesthetist
26.	Intra-Operative Opioid Use	The type and total dose of each opioid used during the operation	Select the option and write total dose for each opiate used.<br>IV Fentanyl (μg)<br>IV Morphine (mg)<br>Intrathecal Fentanyl (μg)<br>Intrathecal Diamorphine (μg)<br>Intrathecal Preservative-Free Morphine (μg)<br>Epidural Morphine (mg)<br>Epidural Fentanyl (μg)<br>Other (specify drug used and dose)	Anaesthetist
27.	Type of General Anaesthetic	The type of general anaesthetic used during the operation	Select one option<br>Volatile<br>TIVA	Anaesthetist
28.	Beat to Beat Blood Pressure Monitoring	If beat to beat blood pressure monitoring was used during the operation e.g. arterial line etc.	Select one option<br>Yes<br>No	Anaesthetist

Field Number	Field Name	Definition	Requirements for data field completion	Suggested person collecting/ providing this data item
29.	Cardiac Output or Stroke volume Variability Monitoring	If cardiac output or stroke volume variability monitoring was used during the operation<br>e.g. LIDCO or other devices to measure pulse pressure variability	Select one option<br>Yes<br>No	Anaesthetist
30.	Central Venous Catheter Sited	If a central venous catheter was sited during the operation	Select one option<br>Yes<br>No	Anaesthetist
31.	Immediate Postop destination planned (pre-op)	The post-operative destination planned for the patient pre-operatively	Select one option<br>ICU<br>HDU<br>Level 1/ POSU<br>Monitored Bed<br>Ward<br>Other (please specify)	Anaesthetist
32.	Immediate Postop destination actual	The actual immediate post-operative destination of the patient	Select one option<br>ICU<br>HDU<br>Level 1/ POSU<br>Monitored Bed<br>Ward<br>Other (please specify)	Anaesthetist
33.	If available, was protocolised postop multimodal analgesia prescribed?	To confirm if protocolised (consistent practice for all patients) multimodal analgesia was used post-operatively. This does not need to be colorectal specific.<br><br>If protocolised multimodal analgesia is not available select Not Applicable	Select one option<br>Not applicable<br>Yes<br>No<br>Unknown	Anaesthetist

Field Number	Field Name	Definition	Requirements for data field completion	Suggested person collecting/ providing this data item
34.	Was the patient seen by the Acute Pain Team on day one post-operatively	Was the patient seen by a member of the Acute Pain Team on day one post-operatively?	Yes<br>No	Anaesthetist
35.	Was ERAS Nurse involved in post-operative care?	Confirm if an Enhanced Recovery (ERAS) nurse was involved in the patient's post-operative care.	Select one option<br>Yes<br>No<br>N/A No ERAS Nurse	Anaesthetist
36.	Length of stay	Total length of stay in hospital for this operation in days	Numeric field<br>Total number of whole days	Surgeon
37.	Surgical Site Infection	Was there any surgical site infection?	Select one option only<br>No<br>Yes, superficial incisional - involving the skin and subcutaneous tissues<br>Yes, deep incisional - involving the soft tissues (fascia and muscle)<br>Yes, Organ/ Space	Surgeon

Field Number	Field Name	Definition	Requirements for data field completion	Suggested person collecting/ providing this data item
38.	Acute Kidney Injury (AKI)	Was there any acute kidney injury classified using the 2012 KDIGO Criteria (modified RIFLE and AKIN)<br><br>https://academic.oup.com/bja/article/115/suppl_2/ii3/272807	Select one option only<br>None<br>Stage 1: Increased serum creatinine (sCr) by 1.5-1.9 times baseline presumed to have occurred in the preceding 7 days or sCr increase ≥0.3 mg dl^{-1} within 48 h or urine output <0.5 ml kg^{-1} h^{-1} for 6–12 h.<br><br>Stage 2: Increased sCr × 2–2.9 or urine output <0.5 ml kg^{-1} h^{-1} for ≥12 h<br><br>Stage 3: Increased sCr × 3 or sCr ≥4 mg dl^{-1} or initiation of RRT or GFR decrease to <35 ml min^{-1} $(1.73\ m)^{-2}$ or urine output <0.3 ml kg^{-1} h^{-1} for ≥24 h or anuria for ≥12 h	Surgeon
39.	Post-Operative Complications	Were there any post-operative complications using Clavien-Dindo definitions<br>Definitions for each grade included in following pages of this guidance document. (page 14)	Select one option<br>None<br>Grade I<br>Grade II<br>Grade IIIa<br>Grade IIIb<br>Grade IVa<br>Grade IVb<br>Grade V	Surgeon
40.	Unplanned return to theatre with this admission	Was there an unplanned return to theatre during this admission?	Select one option<br>Yes<br>No	Surgeon

Field Number	Field Name	Definition	Requirements for data field completion	Suggested person collecting/ providing this data item
41.	Mortality at the point at discharge	The patient's mortality status at the point of discharge	Select one option<br>Alive<br>Dead	Surgeon
42.	ERAS Nurse present at post-operative destination	Was an ERAS nurse present at the immediate post-operative destination identified in field 32?	Select one option<br>Yes<br>No	Surgeon/ Anaesthetist
43.	Started post-operative therapeutic antibiotic	Were post-operative therapeutic antibiotics started?	Select one option<br>Yes<br>No	Surgeon
44.	Discharge destination from hospital	What was the patient's discharge destination following this admission?	Select one option<br>Discharge to usual residence and usual level of support<br>Discharge to usual residence with increased level of support<br>Discharge to destination different from admission as requires additional support<br>N/A patient died	Surgeon
